# The barriers and facilitators to primary care optometrists supporting patients with low vision in England

**DOI:** 10.1111/opo.70026

**Published:** 2025-10-28

**Authors:** Emily Charlesworth, Tarnjit Sehmbi, Michael Bowen, Shahina Pardhan

**Affiliations:** ^1^ Faculty of Health, Education, Medicine and Social Care, Vision and Eye Research Institute Anglia Ruskin University Cambridge UK; ^2^ Centre for Vision Across the Life Span, School of Applied Sciences University of Huddersfield Huddersfield UK; ^3^ The College of Optometrists London UK

**Keywords:** barriers, facilitators, low vision, optometrist, primary care

## Abstract

**Introduction:**

Research suggests that patients with low vision identify optometrists as their core eye care provider within the community; hence, optometrists are well placed to provide support, advice and potentially certify patients. A qualitative study was conducted to ascertain the barriers and facilitators to primary care optometrists supporting patients with low vision.

**Methods:**

An online questionnaire used open‐ended questions to investigate optometrists' knowledge of the Certification of Vision Impairment and registration processes, and the barriers they faced when providing support to patients with low vision. Unmet training needs on low vision and whether optometrists would be happy to certify patients were also explored; data were analysed using deductive thematic analysis.

**Results:**

A total of 148 questionnaires were completed by optometrists in England between October 2023 and January 2024. Varying levels of knowledge were found regarding the certification and registration process. Three barrier‐related themes were identified. These were system barriers, practitioner barriers and patient barriers. Four themes were identified related to facilitators to supporting patients with low vision. These were training methods, training needs, low vision pathway, including optometrists' ability to certify patients and communication between services.

**Conclusions:**

With appropriate remuneration, optometrists reported positively with regard to upskilling and extending their scope of practice to ensure the best care for patients. Optometrists were found to be happy to certify patients if appropriate training and remuneration were received. This would involve the development of a funded Low Vision Pathway in England. Optometrists would benefit from further Continuing Professional Development training with elements from local Eye Clinic Liaison Officers, low vision practitioners and the Hospital Eye Service to improve knowledge around the certification and registration process and local support services available.

**Key points**

- Limited previous research exists on optometrists' knowledge of the certification and registration processes and the barriers they face when supporting patients with low vision in practice.
- Optometrists would benefit from Continuing Professional Development with elements from local Eye Care Liaison Officers, low vision practitioners, the Hospital Eye Service and local support services.
- This study found that optometrists in England would be happy to certify patients if appropriate training and remuneration was received. This could be implemented within a funded low vision pathway.

## INTRODUCTION

In the UK, two million people are estimated to be living with sight loss. From this group in 2024, 322,638 people were registered as sight impaired or severely sight impaired and have received a Certification of Vision Impairment (CVI).^1^ Being certified is optional; therefore, certification numbers are not a measure of sight loss prevalence. A CVI certifies a person as sight impaired or severely sight impaired. This is completed by an ophthalmologist and, with the patient's consent, passed to their General Medical Practitioner (GP), local authority and The Royal College of Ophthalmologists Certifications office.^2^ Certification confirms a patient's vision status to enable them to apply for access to additional social support to improve their wellbeing and independence.^3^ Access to this support from social services has been shown to improve their quality of life.^4^ In addition, those patients who do not yet meet the criteria for a CVI or have declined certification can still access additional support from social services through a Referral of Vision Impairment (RVI).^5^

National data indicate more patients are certified than registered with social services, and not all receive an assessment from their local authority sensory team.^6^ This may be due to a lack of understanding by the patients of the need for both processes. Studies have also suggested optometrists may not be fully aware of the CVI and RVI processes, and so are unable to advise their patients appropriately.^7^ It is possible that some optometrists may have limited knowledge of the different registrations, which would limit the advice they can provide.

Previous studies have highlighted barriers perceived by patients with regard to knowledge and awareness of the available low vision support. This includes awareness of social services and charities,^8^ knowledge around the certification and registration processes,^9^ poor communication with eye care professionals,^10^ lack of resources and inaccessible information about their health and social care needs^11^ and long waiting times for support from social services.^9^ A study published in 2023 found 45% of respondents had not had the certification and registration process explained to them at any stage of their sight loss journey.^12^

Primary eyecare practitioners based in the community can provide the right care and support for their patients, especially given the long waiting times for secondary care and social services support. Research suggests that patients with low vision identify optometrists as their core eye care provider within the community.^13^ Hence optometrists are well placed to provide support and advice. It has been shown that the Primary Care Welsh Low Vision Service provided by trained and accredited optometrists is as effective as a hospital‐based low vision service,^14^ improves access for patients and reduces delays in accessing support.^15^, ^16^

This investigation follows on from a previous study performed in our laboratory using semi‐structured interviews conducted on patients with low vision, Eye Clinic Liaison Officers (ECLOs) and optometrists about their experience of the CVI and registration processes.^7^ The previous investigation found that optometrists had a lack of understanding of the certification and registration process, a lack of clarity about what support can be accessed with certification and registration and mixed messages regarding signposting patients for support. Some optometrists believed it was the responsibility of the Hospital Eye Service (HES), whilst others thought it was the responsibility of the community.^7^ Semi‐structured interviews were only conducted on a limited number of optometrists, and therefore, a national survey is needed to investigate whether these findings are reflected across the profession.

This present study aimed to investigate optometrists' current knowledge of the certification and registration process and the perceived modifiable barriers they face when providing advice and support to patients with low vision. It will further explore their reported training needs in order to be able to provide the best support and inform the development of educational material as Continuing Educational Development. The survey was designed using open‐ended questions to allow for a deeper exploration of topics and real‐world insights.^17^, ^18^

The study also explored optometrists' opinions and thoughts on upskilling and extending their scope of practice to include certifying patients in the future. Optometrists in Wales provide a National Health Service (NHS) low vision service in their practices and are part of the low vision rehabilitation pathway in their area. From 2023, optometrists with the appropriate extra qualifications can complete CVIs for their patients with vision loss due to bilateral dry age‐related macular degeneration (AMD).^19^ This is a new development for optometry in Wales but is not yet available in England. We wished to investigate whether optometrists would be willing to provide this service in their area with appropriate training and remuneration.

## METHODS

The study received ethical approval from Anglia Ruskin University School Research Ethics Panel (MED‐SREP‐ETH2223‐10512 /ETH2324‐2937*). A questionnaire was designed using open‐ended questions to allow qualitative data collection. A qualitative approach was taken to allow the gathering of rich data from optometrists regarding their current knowledge, barriers they face and their opinions of upskilling. This method enabled the gathering of a deeper understanding of the research objectives.^20^ The responses to the questions from the survey were analysed using Thematic Analysis (TA).^21^ A deductive TA approach was taken, as the codes developed reflected the conceptual ideas the researcher aimed to understand through the dataset, which in this case were optometrists' perceived barriers and the training needs to support patients with low vision.^21^ Hence, there was an a priori focus on perceived barriers during the analysis.^21^ Each author was involved in the development of the questionnaire. Two authors were involved in the thematic analysis (EC, TS). EC is an optometrist and therefore understands the role of the optometrist and the barriers they may face in practice. TS is a psychologist who specialises in qualitative research. Codes were identified on a semantic level; therefore, coding mirrored participants' language and concepts.^21^ Coding was used to organise the data into as many headings as necessary to describe the content fully, then similar headings were grouped together to create themes. To ensure rigour^22^ and increase the credibility and dependability of the thematic analysis,^22^, ^23^ two authors (EC and TS) familiarised themselves with and cross‐coded a minimum of 10% of the transcripts. The remaining transcripts were split between these two authors. Any disagreements were discussed until consensus was reached. Each question of the survey was coded separately and will be discussed. Data were collected between October 2023 and January 2024. Information was gathered on practitioners including number of years of experience, gender, work setting (large multiple, small multiple up to 10 branches, independent, hospital, university eye clinic, domiciliary and other), resident or locum practitioner status and any postgraduate qualifications. The questions investigated current knowledge of the registration process, barriers optometrists might have faced when providing support to patients with low vision, barriers to providing support to patients with low vision and any unmet further training needs regarding low vision and low vision services. Completing the survey was taken as implied consent; however, participants had the additional option to consent/not consent for quotes of their responses to be used in the manuscript. The survey questions are included in Table 1.

**TABLE 1:** The questions included in the survey.

What differences are you aware of between the Certificate of Vision Impairment (CVI) and the Referral of Vision Impairment (RVI).
2 What services and charities are you aware of that support people with vision impairment in your area, if any?
3 Where would you signpost patients for further help and support?
4 If you do not signpost patients, please state why.
5 What further training would you require to improve the advice you give to patients with vision impairment?
6 Optometrists in Wales with appropriate extra qualifications can now complete CVI's for patients with vision loss due to bilateral dry AMD. Is this something you would be happy to provide in your area if appropriate training and remuneration was received, please explain why?
7 What is the biggest barrier you face when providing support to patients with vision impairment?

*Note*: A free text response box was used for participants' responses.

Abbreviation: AMD, age‐related macular degeneration.

### Distribution

An online questionnaire and information sheet was developed using the platform *Online Surveys* (jisc.ac.uk/online‐surveys) to follow data laws within the UK. The link was distributed by the College of Optometrists, social media platforms, forums and universities providing an optometry degree. All Local Optical Committees (LOCs) within England were contacted to share the link of the survey with practitioners in their area.

### Patient and public involvement

None.

## RESULTS

A total of 148 optometrists participated. The majority of optometrists worked in either large multiple or independent practices. Thirty‐six percent of responders were male (*n* = 53). The mean number of years qualified was 21.1 (SD 11.5) years. The demographics for the optometrists who completed the survey are shown in Table 2.

**TABLE 2:** The demographics of the 148 optometrists completing the survey.

Gender	
Male	36%
Female	64%
Number of years qualified	21.1 (SD 11.5) years
Practice type	
Independent	39.2%
Large multiple	29.7%
Hospital	13.5%
Small multiple	7.4%
Domiciliary	3.4%
University clinic	1.4%
Other	5.4%

Optometrists were asked about the biggest barrier they faced when providing support to patients with low vision. Following thematic analysis of the open‐ended responses, themes were generated as shown below.

**FIGURE 1:**
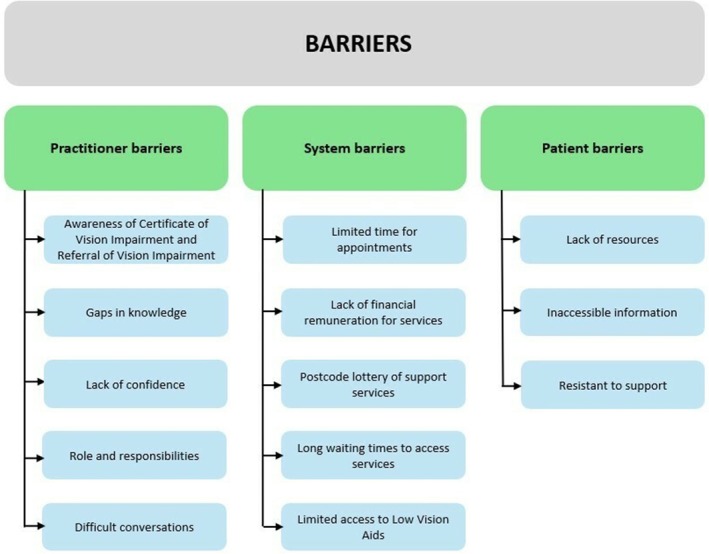
Diagram showing the barrier themes and subthemes found during thematic analysis of the open‐ended responses.

**FIGURE 2:**
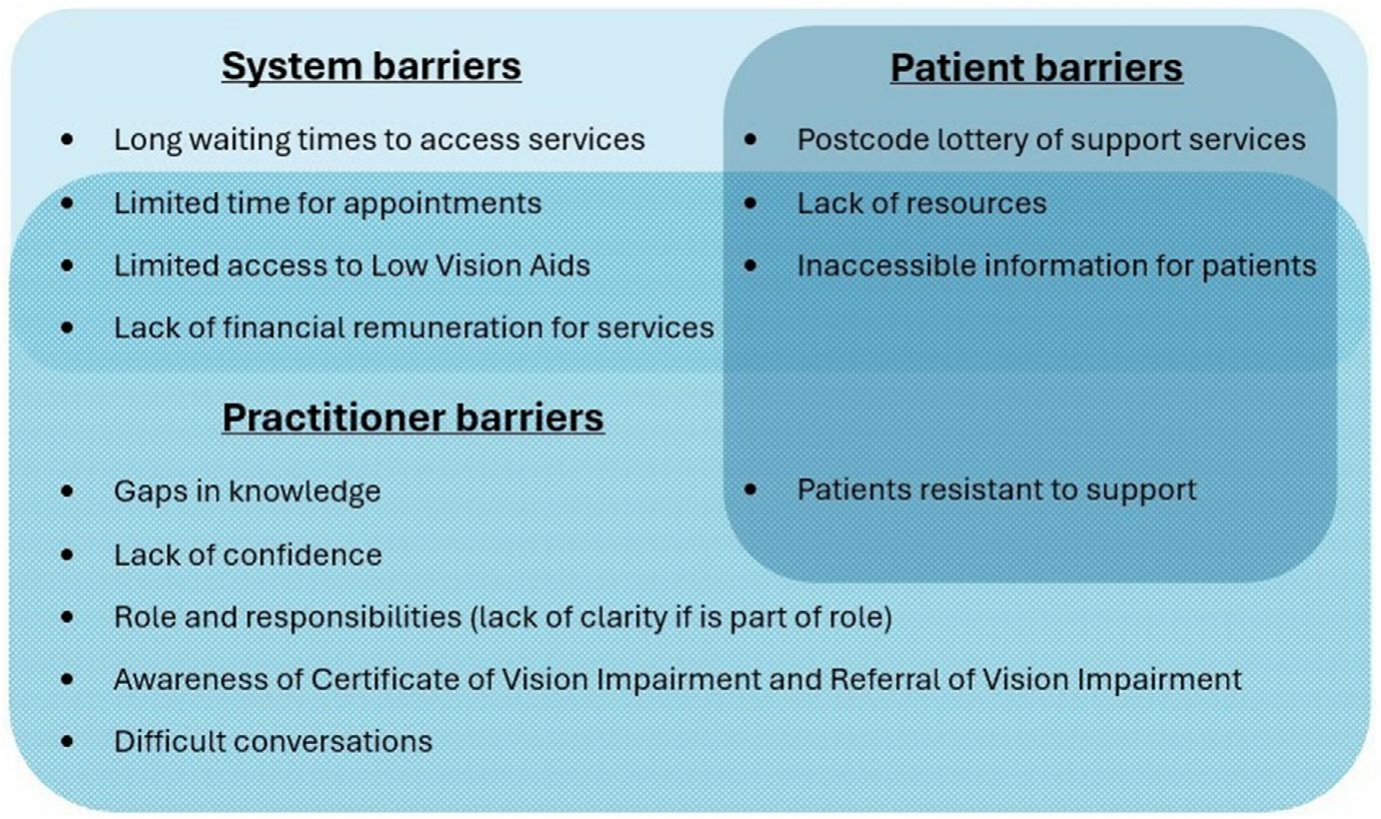
Venn diagram showing the overlap of the system, practitioner and patient barriers that optometrists face when providing support to patients with low vision.

For barriers faced by optometrists, three main themes were generated (Figure 1): (1) practitioner barriers, (2) system barriers and (3) patient barriers. The perceived barriers to providing support to patients with low vision and their overlap are shown in Figure 2.

**FIGURE 3:**
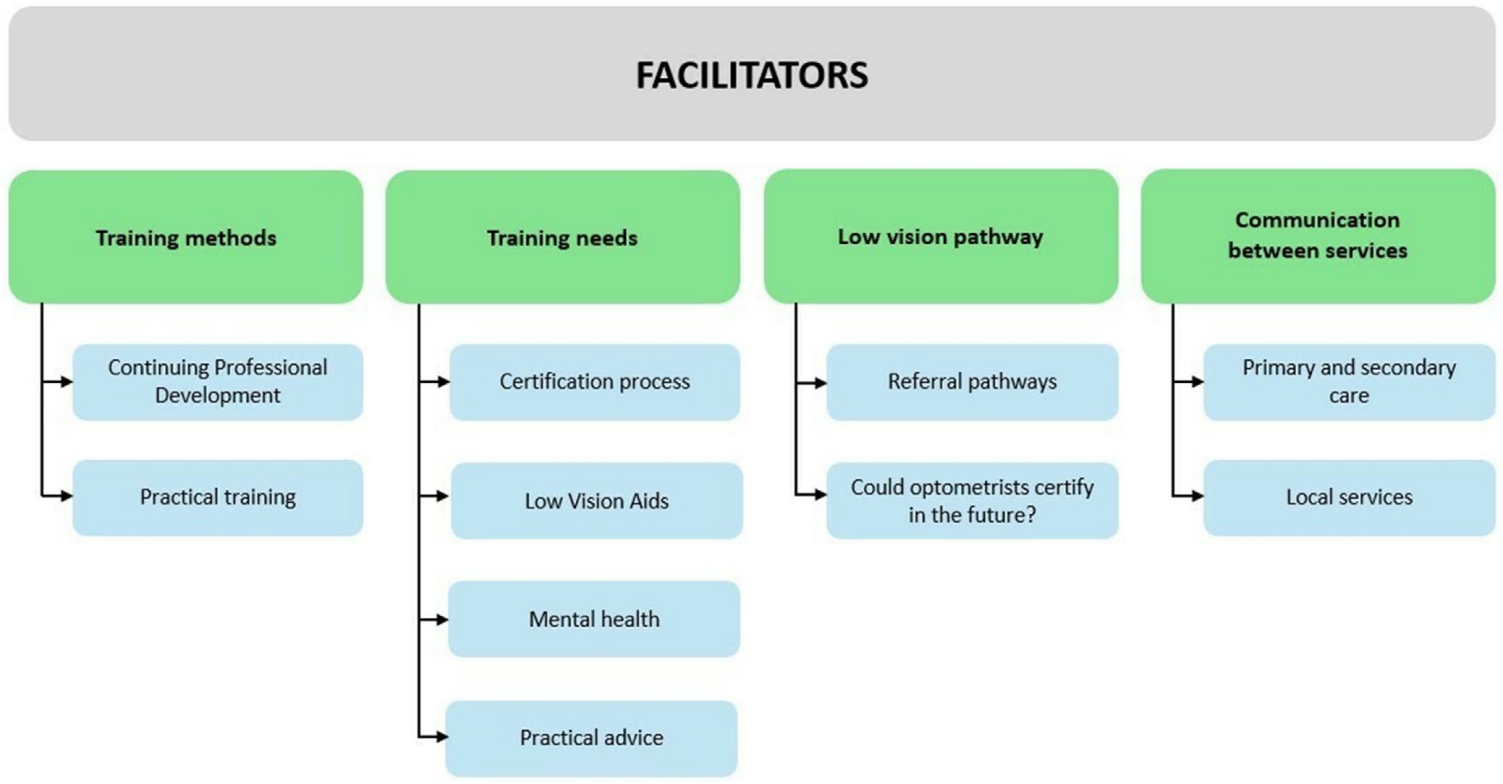
Diagram showing the facilitator themes and subthemes found during thematic analysis of the open‐ended responses.

For identifying facilitators, four main themes were generated (Figure 3): (1) training methods, (2) training needs, (3) low vision pathway and (4) communication between services.

### Barriers

#### Theme 1: Practitioner barriers

##### Awareness of CVI and RVI

Optometrists' response to the question ‘Do you know the differences between CVI and RVI’ showed not all optometrists were aware of the differences between the two types of registrations. Optometrists responded correctly to information on sight impaired (SI)/severely sight impaired (SSI) certification and that certification is completed by an ophthalmologist. (ID refers to the participant's identification number).

CVI issued to patient after being assessed by consultant ophthalmologist to be registered as visually impaired. ID 36

CVI completed by ophthalmologist to register for SSI or SI. RVI is a referral to social services for assistance for daily living needs. ID 18

A CVI registers a patient as officially SI or SSI and can only be completed by an Ophthalmologist. ID 111

There was some confusion regarding RVI, which is voluntary, and receiving a CVI does not automatically register a patient for social services.^2^

Whilst some optometrists responded correctly:

The RVI form is completed to enable a person who is perhaps not meeting the criteria for CVI registration to access VI services in the community. ID 16

The RVI can be issued by an optometrist or other clinical staff for the patient to access help, they don't need to be registered. ID 122

Others showed lower awareness around the process and who is responsible:

RVI is issued at the start of the process of referral. CVI is produced with the consultant at the hospital. ID 53

RVI is a letter to refer a patient for assessment and possible registration, CVI is the form used for registration. ID 174

##### Gaps in knowledge of how to support patients

A practitioner barrier was a gap in knowledge on how to support their patients. Not being up to date on low vision aids (LVAs) information was a concern, as was a lack of clarity on registration pathways and a lack of knowledge of the support and services available to patients.

Not having up to date information of LVAs. Lack of clarity of registration pathways and help available from local authorities, etc. ID 71

No knowledge of local services. ID 59

##### Lack of confidence in how to support patients

Not seeing enough low vision patients in practice was discussed as a cause for lack of confidence. There was found to be a lack of confidence in how to refer patients for certification and registration and a lack of confidence in how to manage these patients in practice.

Unclear on all the pathways (I refer to Ophthalmology for certification if VA is appropriate) but I am not as confident in how to refer to Social Services for example. ID 125

Lack of confidence because I don't see many. Unsure of what low vision aids to advise. ID 77

##### Not geared enough to have difficult conversations

Some optometrists felt they did not know how to broach low vision conversations with patients, with some worried they did not want to come across as too sympathetic, whilst others felt they personally had not done enough to support patients with low vision before referring to other services.

Not feeling as if I have done enough as a practitioner before referring. ID 100

Not knowing how to be empathetic, without being sympathetic. Helping patient to be positive. ID 103

Some optometrists responded that managing expectations around long hospital or social services waiting times was difficult, as they were aware that lengthy wait times are detrimental to patients and make adapting to their vision loss more difficult.

Managing patient expectations. Long waiting lists for home visits, etc. in the community. Depending on the area I have known the waiting list to be up to 2 years. No local provision for housebound patients unable to attend hospital, i.e., no community domiciliary LVA service. Patients attending the LVA service with already very reduced vision which has been progressing for a long time. If they were referred earlier for magnifiers, it would be easier for them to adapt when low magnification is required initially. ID 16

##### Confusion around roles and responsibilities

Some optometrists believe that the responsibility to discuss certification and support lies within the Hospital Eye Service, and this is beyond their role and responsibilities.

Hard to know what support that can be offered. I thought this would be discussed with the hospital eye service. ID 3

The fact that it's somebody else's job. ID 69

Support is from a dedicated health care area. Lack of knowledge on my behalf. ID 37

#### Theme 2: System barriers

##### Limited time for appointments

Optometrists discussed that they did not have the time for these conversations in practice due to short appointment times and busy clinics. Some optometrists felt they were unable to dedicate an appropriate amount of time to their patients with low vision.

Time constraints of a large, busy multiple. ID 36

Being able to allocate enough time in the clinic to dedicate to patients with low vision needs both in the testing room and in the dispensing clinics. ID 64

##### Lack of financial remuneration for services

Optometrists expressed how a lack of a funded low vision pathway restricted optometrists from providing additional support to patients. The current NHS contract does not fund these additional services. The additional time required with these patients would require appropriate remuneration.

Requires much longer chair time but only remunerated the same as non‐VI patients. ID 69

General Ophthalmic Services (GOS) contract does not allow time or financial incentive to truly offer sufficient time and extra training required to make a proper joined up service achievable. As always with the NHS there is too many barriers and too little funding for a proper service to benefit patients. ID 158

Funded time in practice – the GOS is not an adequate vehicle for the space needed to listen to and support people's concerns. Need to offer a magnifier privately. ID 95

##### Postcode lottery of support services

Optometrists mentioned a lack of services for them to refer patients to for further support and described this as a ‘postcode lottery’. Services were described as limited and many were located too far away from the patient to contact. Not all local hospitals provided low vision services to patients. A lack of Eye Care Liaison Officers (ECLOs) and community domiciliary LVA services, non‐responsive social services and a lack of emotional support for the patient were also mentioned:

Postcode lottery of service provision, lack of adequate investment in VRS (Vision Rehabilitation Specialist) services. ID 58

Lack of transparent information about who is able to help with social and mental health issues. ID 163

It's also very difficult to get in touch with the certain departments like eye liaison as they don't answer their phone and don't return calls. ID 131

Limited services available in the area e.g., services/support groups located far away from the patient's home address and the patient being unable to travel to these locations. No local provision for housebound patients unable to attend hospital, i.e., no community domiciliary LVA service. ID 16

The very poor LVA service in ***** (Name of county removed for anonymity). ID 39

##### Long waiting times for patients to access services

Even with the correct knowledge of pathways and services, optometrists saw the long wait times for patients to be seen by secondary care and social services as problematic. The excessive waiting times were seen as a barrier to patients being certified and registered and receiving the support they required. It was mentioned that even after certification and registration, patients had lengthy waiting times before receiving support from social services.

Managing patient expectations. Long waiting lists for home visits etc. in the community. Depending on the area I have known the waiting list to be up to 2 years. Other local hospitals not providing an LVA service which increases the backlog/wait for LVA patients at this hospital. ID 16

Low vision clinics unavailable or long wait in local hospitals. ID 164

##### Limited access to low vision aids

Optometrists commented on a lack of access to LVAs in practice, with some having no access to LVAs. Of the optometrists that did have access, some felt they should not be selling LVAs to patients when they can be provided for free by the NHS. It was the understanding of a few optometrists that patients cannot access LVAs without a CVI.

The cost of patients then having to pay privately for any LVAs that I supply. ID 83

We are a private practice now and do not receive the NHS payment for seeing sight impaired patients and remuneration for LVA supplied. ID 104

#### Theme 3: Patient barriers

##### Lack of resources

Optometrists commented on a lack of resources for patients with low vision. It was suggested that being able to provide all the relevant information in one information pack could benefit patients, including local contact information for social services, charities and mental health support.

Have a guide which can specify exactly what social services, charities are available for people with vision impairments. Simple patient leaflets with all relevant contacts for social and mental health help available. ID 163

##### Inaccessible information for patients

Some optometrists reported that when they had resources available to give to patients, such as letters, leaflets and websites, they were inaccessible for many with low vision, but no alternatives were available due to a lack of resources.

A lot of help is online which is obviously difficult for the patient to access. Even when I give them a leaflet, it would be great to see them again for a follow up. ID 63

It feels like we're constantly signposting patients to different places and giving this information on paper that they cannot read. ID 160

##### Resistant to support

Optometrists discussed how patients can struggle to come to terms with their sight loss and the loss of independence. Patients being resistant can be a barrier to providing support. It was discussed how encouraging patients to bring family or friends to their appointments can help to promote discussion around the help and support needed.

Understanding of the patient, I try and explain the sight loss, the use of low vision aids and the working distance but patients themselves give the resistance and just want new glasses. ID 98

I find older patients wish to remain independent but are not receptive to having help from sight support services. I don't know if it's a generational issue, an attitude of ‘I can manage’ and ‘I don't want to make a fuss.’ I find it helps to have family members present when discussing getting help at home. ID 135

### Facilitators

Four themes were generated when identifying facilitators. These are shown in Figure 3.

#### Theme 1: Training methods

##### Continuing professional development

Optometrists' responses included the need for further training with Continuing Professional Development (CPD) courses.

More low vision CPD. ID 84

Much more CPD. ID 55

And this needs to be in the form of webinars, workshops, articles and lectures. Ongoing CPD on low vision topics was discussed and requested to be interactive to enable questioning:

Webinars, interactive session where we can ask questions. ID 121

Clearly written CPD article. ID 127

Local ECLOs, low vision practitioners, the local HES or Local Optical Committee would be best placed to conduct this formal training as pathways and services are likely to differ in different locations.

Perhaps a refresher lecture organised by our LOC to remind us of LVL, RVI, CVI, ECLO and local organisations and contacts for providing support? ID 109

Several optometrists highlighted that they would be interested in studying for a higher qualification such as the College of Optometrists' Professional Certificate in Low Vision to improve their knowledge and management of patients with low vision.

Further training through the Higher Certificate in Low Vision. ID 50

##### Practical training

Alongside CPD training, numerous optometrists felt they would benefit from more practical training. It was discussed that this could bring awareness to what low vision clinics can offer, what optometrists can do to maximise a patient's time in primary care and help to keep optometrists updated on local services.

I would like to have the opportunity to sit in with the ECLO service periodically to keep updated. ID 129

Awareness of what HES LV clinic can offer and what optometrists can do in primary care to maximise patients time in this clinic. ID 117

#### Theme 2: Training needs

##### The registration process

Optometrists requested training on several topics, including the registration process and the vision requirements to qualify as SI/SSI and enable registration for a CVI.

Understanding the criteria for visual impairment, e.g., dry AMD may have reduced VA but not a constricted field – does that qualify? ID 77

##### Low vision aids

Further training on LVAs is required. Many optometrists wish to be able to provide NHS‐funded magnifiers and LVAs to patients in practice to reduce pressures on the HES and avoid lengthy waiting times for the patient.

More low vision assessment training and assessing for magnifiers. ID 35

I'm not sure about LVAs for working age people, such as computerised systems. ID 136

What would also help would be to be able to give out magnifiers funded by the NHS. Currently I charge for these or refer to HES at huge system cost and avoidable pressure. ID 134

Assessing for magnifiers – or some ability to issue magnifiers through the NHS. ID 35

##### Mental health

Some optometrists felt they should receive training on other aspects of care such as mental health support for patients as this is an important part that can be overlooked. With the appropriate training, they felt they would be in a suitable position to signpost patients when needed.

Mental support – not much on this, more on physical help with improving their remaining vision. ID 151

What we can do regarding sign posting and what advice we can give to cover other aspects of care such as mental health support. ID 161

##### Practical advice

Training on practical advice for patients was highlighted. Assessing patients in the testing room does not always address the problems they are experiencing at home. Therefore, providing more practical advice and non‐optical solutions such as improvements around the home, information on lighting, technology and navigating outdoors would be beneficial.

Training on how daily activities could be improved with non‐optical corrective measures. ID 68

Advice on how to give support for navigating around the home and outdoors. ID 64

Improvements around the home, e.g., lighting etc. ID 50

#### Theme 3: Low vision pathway

##### Referral pathways

Optometrists requested information on standard referral pathways and protocols to be able to advise patients appropriately. Some suggested a national mailing list to alert eyecare practitioners (optometrists, ophthalmologists, ECLOs, dispensing opticians, etc.) to relevant changes in pathways and services that would benefit patients.

How to refer someone into pathways for registration. ID 123

Some sort of national mailing list with updates about services would be useful to be able to sign up to. ID 43

It would be a miracle to see a cohesive step by step pathway. It would be good to know who did what and how to access their help. ID 53

##### Optometrists certifying in the future

Optometrists in Wales with appropriate extra qualifications can now complete CVIs for patients with vision loss due to bilateral dry AMD. These optometrists are part of a low vision rehabilitation pathway and provide NHS low vision services in their practices. Optometrists were asked if they would be happy to provide CVIs for patients in their area if appropriate training and remuneration were received. Optometrists were positive about upskilling and extending their scope of practice to include this. Optometrists felt they were able to provide patients who met the requirements of registration with a CVI. Many believed this would benefit the HES and patients. It would help to reduce the burden on the HES, avoid patients having lengthy waiting times and remove the difficulty of attending an appointment with an ophthalmologist for registration.

I think allowing specially certified optometrists to be able to register patients with CVI instead of having to see an ophthalmologist would reduce the burden on eye clinics. ID 135

This would be amazing because there is such a long wait just to see an ophthalmologist and get registered, and often patients have to make a special (and difficult) trip to hospital just to be registered. ID 141

Although the majority of optometrists shared positive opinions, some had concerns regarding the reluctance of optometrists completing CVIs in the future. These included:

Appropriate remuneration: Whilst optometrists expressed that they thought this was a good idea and a step in the right direction, several highlighted this would only be possible with appropriate remuneration.

Only if there is financial support as well. ID 99

Lack of time was expressed as a concern.

Lack of time in busy clinics. ID 101

Whilst another expressed a lack of confidence.

I would not feel confident to do that. ID 1

I have had patients argue with me over whether their vision loss should qualify them to be registered sight impaired and it is not a conversation I wish to invite repeatedly. ID 33

There was a concern that this is stepping into the domain of ophthalmologists and is not part of the optometrists' role. Some thought this was best discussed in a hospital setting.

I don't think ophthalmologists would trust us to do this. ID 151

There were concerns about patients missing out on new techniques and procedures if they are not seen by an ophthalmologist:

I think it should remain with ophthalmologists; patients may miss out on new techniques and procedures if they're not seen. ID 89

Patients may lose out if strict criteria are applied as some ophthalmologists can use their discretion and still list patients who do not meet the strict criteria.

Sometimes patients don't hit the criteria for CVI but the consultants can use their discretion and still list them. I feel it's not always black and white. Some vision loss affects people differently. ID 129

#### Theme 4: Communication between services

##### Primary and secondary care

Optometrists expressed a need for improved communication between both primary and secondary care to enable all practitioners to be aware of each other's involvement in a patient's sight loss journey.

The NHS can be very bitty. I think it's often the case that one service doesn't know what others do and sometimes aren't even aware of each other. I think just being aware of who is where and what they offer would be great. ID 6

##### Local services

As discussed earlier, a barrier to support from low vision services can be due to a ‘postcode lottery’ of services available in a patient's local area. As each local area can have a wide variety of different support and services available to patients, it is important that optometrists have access to local information. This includes improving communication channels with local accessible services and keeping up to date relevant contact information and services available so that patients and family members can be signposted correctly.

I am more aware of national organisations than local ones, which may not be as useful for patients seeking help. More awareness of local availability of help would be beneficial. ID 103

Better access to up to date local information and where to find it. ID 27

Local charities and support groups reaching out to make us aware of what they can offer. ID 35

## DISCUSSION

This study sought to investigate the barriers optometrists face when providing support to their patients with low vision. Deductive thematic analysis generated three barrier‐related subthemes: system, practitioner and patient barriers. Four themes were generated when identifying the facilitators, that is, training methods, training needs, the low vision pathway and communication between services.

### Barriers

The system barriers found were limited time for appointments, lack of financial remuneration for services, postcode lottery of support services, long wait times to access services and limited access to LVAs. Some of these barriers were found to have significant overlap, which subsequently impacted the optometrists' ability to help patients with low vision, as well as the care and advice that these patients received along the eye care pathway. Many of the barriers identified by optometrists could be addressed by the implementation of a low vision pathway where appropriately trained optometrists provide funded NHS low vision services in their practices with appropriate remuneration. The current GOS NHS contract for optometrists in England does not provide remuneration to provide support for patients with low vision. Appropriate remuneration would address the identified barrier of a lack of financial remuneration for services. Implementation of a low vision pathway would also enable optometrists to spend adequate time with patients, thereby addressing the limited time for appointments barrier. This would enable optometrists to provide support within primary eye care services.

A postcode lottery of support services was found to be impacting low vision services in England. Variability and inconsistencies in support for people with low vision have been shown, as well as lengthy waiting times to access secondary care services. Some areas were found to have limited services for optometrists to refer patients to. Not all local hospitals offer low vision services or have an ECLO, meaning patients had to travel to access these services or did not access these services at all. Access to services has previously been shown to be dependent on individuals seeking support themselves, rather than service providers having a proactive role. Patients should not be left to navigate the support systems available, and this needs to be addressed.^9^ NHS trusts are different organisational units within the NHS, which manage NHS‐funded healthcare services. They deliver a range of services either by geographical location or by specialised function. It has been shown some Trusts have no state‐funded low vision provision for either children (17%), adults (10%) or both (21%),^24^ and a lack of equality in accessing support, which varies with location.^25^ Updated mapping of low vision services across the UK will help to identify these gaps and indicate where areas for improvement exist.

Limited access to LVAs was another system barrier identified. Some optometrists felt it was unethical to sell LVAs and magnifiers when patients can be issued such appliances by the NHS in secondary eye care services. A funded pathway could enable optometrists to supply NHS‐funded magnifiers within primary care optometry, reducing waiting times for patients and bringing care closer to home. Such pathways exist in Wales where patients can see an accredited Low Vision Service Wales practitioner within their local high street practice. Services are free of charge for the patient, including magnifiers, lamps, tinted spectacles, specialist advice and referral for other support. Optometrists then receive a clinical fee for providing this service.^26^

Patient barriers were a lack of resources and inaccessible information. Optometrists discussed how the resources to which they had access, such as leaflets and websites, may be inaccessible to patients with sight loss. Beverly et al. found patients received varying levels of information, and this was generally given verbally.^11^ Many patients sought additional information by themselves.^11^ The development of accessible resources for optometrists to provide to patients is required. Patients with low vision need to be involved in the development of resources to ensure the information is accessible^27^ and available in different formats.^28^

Awareness of CVI and RVI, gaps in knowledge and a lack of confidence were found to be practitioner barriers. However, this study identified the training needs of optometrists to address these, as discussed below. Optometrists were positive about upskilling and extending their scope of practice.

### Facilitators

This study identified the facilitators to optometrists supporting patients with low vision. The needs identified could inform the development of appropriate training for optometrists. Optometrists felt they would benefit from more formal training in the form of CPD lectures, workshops, webinars and practical training, which are interactive to allow for questioning. These educational materials would benefit from input from ECLOs, low vision practitioners, the HES and patients. Patient and Public Involvement is an integral part in shaping health and social care services^29^ and in the development of educational materials for health professionals to promote patient‐centred practice.^30^ Educational events regarding referral pathways, low vision services, social services support and mental health support may benefit from including elements from each region as these are likely to differ by location. Optometrists requested increased training on the certification process and registration process. Whilst optometrists were found to have a good awareness of the CVI process, gaps in knowledge and confidence were found when providing low vision advice to patients. Through the generation of subthemes, it was determined that optometrists had much more confusion around RVI and registration to social services, with some admitting they did not know what an RVI would be used for. Others incorrectly believed an RVI was used to refer patients for CVI registration. Who completes an RVI was also found to be an area of confusion, and this should be addressed.

Further training needs were identified on optical interventions such as LVAs, as well as non‐optical interventions including practical advice that optometrists can provide to patients. Optometrists also raised concerns about managing conversations around mental health and how to initiate difficult conversations regarding sight loss, particularly if the patient was resistant to support. Good communication skills are required to ensure patients are treated respectfully, to investigate their needs appropriately and manage expectations. Communication has previously been found to be a barrier to referral to low vision services by both practitioners and patients.^31^ Patient interactions with clinicians can have a lasting impact on how well a patient is able to come to terms with their visual impairment^10^; therefore, optometrists would benefit from further training in this area.

It was determined that optometrists would benefit from increased knowledge of referral pathways and the low vision services available to patients. The present study found many optometrists were unaware of local low vision services or what support they can provide for patients. Previous studies have discussed the poor utilisation of low vision services, including a lack of awareness of services and charities and what they provide for patients,^8^ a lack of communication about low vision services to patients^8^ and a lack of knowledge of services amongst ophthalmologists.^32^ Whilst most optometrists can name large national sight loss charities, many were unaware of local services and requested more information on this. Future training for optometrists should include elements relevant to their practising area to enable them to be better informed of and develop links with local services.

The final facilitator identified was enabling optometrists to certify patients. Optometrists were positive about upskilling and extending their scope of practice to include this ability if appropriate training and remuneration were received. This would benefit patients by reducing lengthy waiting times in secondary eye care and allow for earlier intervention and timely certification.^33^ It would also enable improved accessibility for patients.^34^ Barlett et al. found that patients supported the provision of CVI within primary eye care and identified the optometrist as their core eye care provider.^13^ The decision to certify is a decision‐making process involving quantitative measures, subjective assessment and clinical judgement.^35^ It has been shown that low vision optometrists and ophthalmologists demonstrate a comparable agreement on certification,^33^ and importantly, ophthalmologists support the idea of optometrists certifying patients.^36^ When the present questionnaire was conducted, optometrists in Wales could complete CVI's for patients with sight loss due to bilateral dry AMD. Due to its success, this service has been extended since June 2025 to allow optometrists to certify patients with sight loss due to any ocular condition.^37^ A similar funded pathway in England could be an appropriate step forward in supporting patients with vision loss, bringing care closer to home, reducing both the pressures on the HES and waiting times for patients. Stolwijk et al. found half of patients felt they would have benefited from receiving information sooner.^31^ A funded pathway would allow optometrists to offer support to patients earlier in their sight loss journey, reducing the need for referral into secondary eye care services.

### Limitations

As the respondents to the survey were self‐selecting, the optometrists contributing to the findings may have had a greater interest in low vision and therefore may not represent the views of all optometrists in England. In an attempt to receive a wide array of responses, the survey was shared with every Local Optical Committee in England. The workplace setting proportions of respondents were similar to the latest General Optical Council registrant survey; however, optometrists who work in national chains were under‐represented.^38^ The survey was designed using open‐ended questions to allow for a deeper understanding of topics, although the level of detail provided by each respondent varied.

## CONCLUSION

Ophthalmology departments have been shown to be the busiest outpatient department within the NHS, facing significant capacity pressures, with most NHS trusts short of consultant level care.^39^ The development of a funded low vision pathway in England would help to reduce the current inequalities in the accessing and provision of low vision services. Patients would be able to access support sooner in their sight loss journey, have access to care closer to home, and the pathway would help to reduce pressures on secondary eye care services. The present study found that optometrists were positive about upskilling and extending their scope of practice to ensure the best care for patients. Optometrists would benefit from formal CPD training. Due to the variety of support and services across the UK, it is essential that elements of training include information on the optometrist's local pathways, services and support services.

## AUTHOR CONTRIBUTIONS

**Emily Charlesworth:** Conceptualization (equal); data curation (lead); formal analysis (lead); investigation (lead); methodology (equal); project administration (lead); validation (equal); visualization (equal); writing – original draft (lead); writing – review and editing (lead). **Tarnjit Sehmbi:** Data curation (supporting); formal analysis (supporting); writing – original draft (supporting); writing – review and editing (supporting). **Michael Bowen:** Data curation (equal); writing – original draft (supporting); writing – review and editing (supporting). **Shahina Pardhan:** Conceptualization (equal); data curation (supporting); formal analysis (supporting); investigation (equal); methodology (equal); supervision (lead); writing – original draft (supporting); writing – review and editing (supporting).

## FUNDING INFORMATION

Anglia Ruskin University Post Doctoral Research Funding for the Vision and Eye Research Institute.

## CONFLICT OF INTEREST STATEMENT

The authors declare no conflict of interest associated with this manuscript.

## Data Availability

The data that support the findings of this study are available from the corresponding author, EC, upon reasonable request.
